# Comparative genomic study of *ALDH* gene superfamily in *Gossypium*: A focus on *Gossypium hirsutum* under salt stress

**DOI:** 10.1371/journal.pone.0176733

**Published:** 2017-05-10

**Authors:** Yating Dong, Hui Liu, Yi Zhang, Jiahui Hu, Jiyu Feng, Cong Li, Cheng Li, Jinhong Chen, Shuijin Zhu

**Affiliations:** Department of Agronomy, Zhejiang Key Laboratory of Crop Germplasm, Zhejiang University, Hangzhou, China; Nanjing Agricultural University, CHINA

## Abstract

Aldehyde dehydrogenases (ALDHs) are a superfamily of enzymes which play important role in the scavenging of active aldehydes molecules. In present work, a comprehensive whole-genomic study of *ALDH* gene superfamily was carried out for an allotetraploid cultivated cotton species, *G*. *hirsutum*, as well as in parallel relative to their diploid progenitors, *G*. *arboreum* and *G*. *raimondii*. Totally, 30 and 58 *ALDH* gene sequences belong to 10 families were identified from diploid and allotetraploid cotton species, respectively. The gene structures among the members from same families were highly conserved. Whole-genome duplication and segmental duplication might be the major driver for the expansion of *ALDH* gene superfamily in *G*. *hirsutum*. In addition, the expression patterns of *GhALDH* genes were diverse across tissues. Most *GhALDH* genes were induced or repressed by salt stress in upland cotton. Our observation shed lights on the molecular evolutionary properties of *ALDH* genes in diploid cottons and their alloallotetraploid derivatives. It may be useful to mine key genes for improvement of cotton response to salt stress.

## Introduction

Endogenous aldehydes molecules are intermediates in common metabolic pathways [1]. However, excess aldehydes are toxic and deleterious to organism since the reaction of their carbonyl group with cellular nucleophiles. Aldehyde dehydrogenases (ALDHs; enzyme class EC: 1.2.1.3), considered as ‘aldehyde scavengers’, can metabolize a variety of aromatic and aliphatic aldehydes to their corresponding carboxylic acids by irreversible oxidation [2, 3]. ALDHs comprise a gene superfamily which are evolutionarily conserved and have been found in both prokaryotes and eukaryotes [4]. According to the criteria established from ALDH Gene Nomenclature Committee (AGNC), ALDHs can be divided into 24 families throughout all taxa [3]. Plant species contain 14 distinct families including ALDH2, ALDH3, ALDH5, ALDH6, ALDH7, ALDH10, ALDH11, ALDH12, ALDH18, ALDH19, ALDH21, ALDH22, ALDH23, and ALDH24. Among them, families ALDH10, ALDH12, ALDH19, ALDH21, ALDH22, ALDH23, and ALDH24 are unique in plant kingdom. To date, genome-wide analysis of *ALDH* gene superfamily were performed in many plant species. There were 16 *ALDH* genes in *Arabidopsis* [5], 20 in rice [6], 18 in soybean [7], 26 in *populus* [8], and 23 in maize [9], etc.

Since the first identified plant *ALDH* gene *rf2* was reported to function in male fertility of maize [10], previous studies have demonstrated that *ALDH* genes are involved in various metabolic and molecular detoxification pathways. Plant *ALDH* genes are induced under wide range of abiotic stresses such as drought, cold, high salinity, and heavy metals which indicated their potential role in improvement of plant stress tolerance. It has been proved that ALDH7A1 is a novel enzyme that involved in cellular defense against hyperosmotic stress [11]. Overexpression of *ALDH3I1* in *Arabidopsis* could enhance the plant’s tolerance to many stresses [12]. Whereas, *OsALDH11* and *OsALDH22* were highly reduced by drought stress in rice [6]. What’s more, it was reported that transferring the *TraeALDH7B1-5A* of wheat into *Arabidopsis* conferred significant drought tolerance in transgenic plants [13]. In addition, ectopically expressing the soybean antiquitin-like *ALDH7* gene in *Arabidopsis* and tobacco resulted in improvement of tolerance towards drought, salinity, and oxidative stress [14]. Though *ALDH* gene superfamily has been reported in *G*. *raimondii* [15], little is known about their detail information in other cotton species, especially the potential role under salt stress in upland cotton.

Cotton is one of the most important economic crop worldwide. There are approximately 50 cotton species in *Gossypium* genus, among which there are four cultivated species. They include two diploids, *Gossypium arboreum* (A_2_) and *G*. *herbaceum* (A_1_), and two natural allotetraploids, *G*. *hirsutum* (AD_1_) and *G*. *barbadense* (AD_2_). Compared with wild species, the cultivated ones are able to produce economically valuable fibers. It has been proved that allotetraploid cottons were diversified from the same polyploidization events nearly 1–2 million years ago [16]. In addition, the genomes of *G*. *arboreum* and *G*. *raimondii* (D_5_) were considered to be the potential donors of A-subgenome and D-subgenome of the two allotetraploid cotton species, respectively. Recently, the four cotton species have been sequenced completely [17–22]. *G*. *hirsutum* accounts for over 90% of commercial cotton production globally and is an ideal model for polyploidy research. As a kind of pioneer crop in saline-alkali, the molecules and mechanisms related with salt stress response are still remain to be uncovered. As mentioned above, ALDHs are proposed to play an important role in plants under abiotic stress. The publications of genome sequences data of these four cotton species give us an access to investigate *ALDH* gene superfamily systematically in *Gossypium*, and mine key genes for improvement of plant salt tolerance.

In this study, comparative genomics approaches were applied to analyze *ALDH* gene superfamily in *G*. *hirsutum* and its diploid progenitors *G*. *arboreum* and *G*. *raimondii*. At the same time, the other cultivated allotetraploid cotton species *G*. *barbadense* was also adopted for systematic evolution investigation. The potential roles of *ALDH* gene superfamily in *G*. *hirsutum* response to salt stress were highlighted. The genetic structure and evolutionary relationship analyses were carried out, and the tissue-specific expression profile of *ALDH* gene superfamily in *G*. *hirsutum* was generated. Our results provided insights into the evolutionary processes of polyploidization with *ALDH* gene superfamily as an example, and associated the genomic substructures for the improvement of cotton tolerance to salt stress.

## Materials and methods

### Database search and sequence retrial for ALDH proteins

The completed genome sequences of four cotton species *G*. *arboreum* [21], *G*. *raimondii* [17], *G*. *hirsutum* acc. TM-1 [20] and *G*. *barbadense* cv. Xinhai21 [22] were downloaded from CGP (http://cgp.genomics.org.cn/), Phytozome (http://phytozome.jgi.doe.gov/pz/portal.html#!info?alias=Org_Graimondii), (http://mascotton.njau.edu.cn) and (http://database.chgc.sh.cn/cotton/index.html), respectively. The published ALDH proteins of *Arabidopsis* [5] and rice [6] were obtained from TAIR (http://www.Arabidopsis.org/) and MSU (http://rice.plantbiology.msu.edu/), respectively. Afterwards, the ALDH proteins from *Arabidopsis* and rice were used as queries to search against those cotton genome databases with BlastP and tBlastN program with a stringent E value cut-off (≤e−20). Then, all hits were subjected to Pfam (http://pfam.sanger.ac.uk/) [23] and NCBI Conserved Domain Database (http://www.ncbi.nlm.nih.gov/cdd) [24] to confirm the presence of the conserved domain. Interproscan (http://www.ebi.ac.uk/Tools/pfa/iprscan/) program [25] was subsequently applied to determine each candidate member of ALDH protein superfamily. The retrieved sequences possessing motifs Pfam00171 (ALDH family), PS00687 (ALDH glutamic acid active site), PS00070 (ALDH cysteine active site), KOG2450, KOG2451, KOG2453, and KOG2456 (all aldehyde dehydrogenase) were retained for further analyses. To characterize the members of *G*. *hirsutum* ALDH superfamily, the pI and molecular weight of the full-length proteins were calculated by Compute pI/Mw tool from ExPASy (http://web.expasy.org/cgi-bin/compute_pi/pi_tool) [26]. And the CELLO v2.5 (http://cello.life.nctu.edu.tw/) [27] was applied to predict the subcellular localization.

### Phylogenetic analysis and genomic organization prediction

For phylogenetic analysis of all the putative ALDH proteins, multiple sequence alignments were created using ClustalX 2.0 [28] with default option, followed by adjustment and refinement with BioEdit V7.2.5 [29]. Then, phylogenetic trees were constructed with MEGA 5.2 software [30] using a neighbor-joining (NJ) method. The parameters were as follows: poisson correction model, pairwise deletion and bootstraps test with 1000 replicates for statistical reliability. Furthermore, maximum likelihood (ML) analysis with PhyML software [31] was applied in the tree construction to test the reliability of NJ method.

The structures of *ALDH* genes were parsed from respective genome files, and portrayed graphically using the online program Gene Structure Display Server (http://gsds.cbi.pku.edu.cn/) [32].

### Chromosomal location and gene duplication

To map the location of *ALDH* genes in *G*. *hirsutum*, the chromosomal distribution of *ALDH* genes were illustrated by Circos software [33] according to their positional information provided in the genome files. Two types of *ALDH* gene duplication events were identified within the *G*. *hirsutum* genome. Only the length coverage covered > 80% of the longer one between aligned gene sequences and the similarity of the aligned regions was > 80% can be defined as duplication events [34–36]. Referring to different chromosomal location, they can be designated as tandem duplication or segment duplication. PAL2NAL v14 [37] was then run on these full-length *ALDH* gene pairs to calculate the nonsynonymous substitutions rate (dN) and synonymous substitution rate (dS) of evolution. The ratio of dN to dS (dN/dS) were then assessed to determine the selective pressure of duplicate genes [38,39].

### Plant materials, growth condition, and salt treatments

One-week-old seedlings of the upland cotton genetic standard line, G. *hirsutum* acc. TM-1, were transplanted into polypots (10 cm in diameter) with full-strength Murashige and Skoog (MS) medium and transferred to growth chamber with temperature of 28°C, relative humidity of 60%, and photoperiod of 16 hours light and 8 hours dark. At the appearance of the true leaf, the seedlings were subjected to salt treatment by transferring them to a MS medium with additional 0, 100, 150 and 200 mM NaCl, which represented the control condition, slight stress, moderate stress, and severe stress, respectively. Three biological replicates were conducted for each sample. After treatments for two weeks, the root, stem, cotyledon and leaf were harvested from each individual, immediately frozen with liquid nitrogen and then stored at -80°C for RNA isolation.

### RNA isolation and expression analysis of *ALDH* genes

Fifty-eight pairs of *ALDH* gene specific primers from *G*. *hirsutum* were used to study the expression profile of *ALDH* gene superfamily by qRT-PCR. Total RNAs of all the collected samples were isolated using EASYspin Plus RNAprep Kit (Aidlab, Beijing, China). A NanoDrop 2000 Spectrophotometer (NanoDrop Technologies, Wilmington, DE, USA) was used to detect the quantity and quality of total RNAs. Approximately 500 ng RNA was reverse transcribed using the PrimerScript 1st Strand cDNA synthesis kit (TaKaRa, Dalian, China) to synthesis cDNA. All the protocols were followed the manufacturer’s instructions. qPCR was performed with Lightcycler 96 system (Roche, Mannheim, Germany) using SYBR the premix Ex taq (TakaRa, Dalian, China) in 20 μL volume according to the supplier’s protocols. The specific primers used in current research are listed in S1 Table. *G*. *hirsutum UBQ7* was used as internal control to normalize all data. Each gene was run in triplicate from three biological replicates. 2^−ΔΔCt^ method was carried out to calculate the relative expression levels [40]. And the heatmap for expression profiles were generated with the Mev 4.0 software [41].

## Results

### Characterization of upland cotton *ALDH* gene superfamily

The completed genome sequencing of cotton species, *G*. *arboreum* (A_2_), *G*. *raimondii* (D_5_), *G*. *hirsutum* (AD_1_), and *G*. *barbasense* (AD_2_) resulted in the whole-genome exploration of *ALDH* gene superfamily in *Gossypium*. In this study, 30, 30, 58, and 58 non-redundant ALDHs encoding members of 10 *ALDH* gene families (ALDH2, ALDH3, ALDH5, ALDH6, ALDH7, ALDH10, ALDH11, ALDH12, ALDH18, ALDH22) were identified respectively in the aforementioned four *Gossypium* (S2 Table). The nomenclature and description of *ALDH* genes in the four *Gossypium* species were referred as the criteria established by the ALDH Gene Nomenclature Committee (AGNC). According to the AGNC criteria, deduced cotton ALDH sequences with greater than 40% identical to other previously identified ALDH sequences composed a family, sequences with less than 40% identical would form a new ALDH protein family. For sequences that were more than 60% identical, they were grouped as a protein subfamily. To classify each protein family based on AGNC, all the ALDH proteins from *G*. *arboreum*, *G*. *raimondii*, *G*. *hirsutum*, and *G*.*barbadense* were designated as GaALDH, GrALDH, GhALDH, and GbALDH, respectively. The proteins belonged to different families were followed by the family designation number (1, 2, 3, 4, etc.), and subsequently by a subfamily designation letter (A, B, C, D, etc.). Finally, an individual gene number was added according to chromosomal order within each subfamily. Moreover, A and D were assigned to distinguish genes from A- and D-subgenome of allotetraploid cotton species.

As illustrated in Table 1, family 2 was the largest one with 15 *ALDH* genes in allotetraploid cottons and eight in diploid cottons, respectively. Families 5, 7, 12 and 22 were the smallest, with only one representative in the diploid progenitors. Compared to other well characterized plant *ALDHs*, *G*. *hirsutum* and *G*. *barbadense ALDH* gene superfamilies were the most expanded ones with 58 members. In *G*, *hirsutum*, these candidate *ALDH* genes encoded proteins ranging from 33 kDa (GhALDH2C3D) to 124 kDa (GhALDH6B3D). And the other detailed information of *G*.*hirsutum* ALDH proteins such as the length, isoelectric points (pI), and the predicted subcellular localization were listed in Table 2.

**Table 1:** The number of *ALDH* gene superfamily members identified in *Gossypium*.

Family	*G*. *arboreum*	*G*. *raimondii*	*G*. *hirsutum*	*G*. *Barbadense*
Family 2	8	8	15	15
Family 3	5	6	12	11
Family 5	1	1	2	2
Family 6	4	3	6	6
Family 7	1	1	1	2
Family 10	2	2	4	5
Family 11	3	3	6	5
Family 12	1	1	2	2
Family 18	4	4	8	8
Family 22	1	1	2	2
Total	30	30	58	58

**Table 2:** The information of *ALDH* gene family in *G*. *hirsutum*.

Family	Gene Name	Gene identifier	Chromosome	Genomics position	CDS	Size (AA)	*Mw(kDa)*	*pI*	Subcellular Localization	Strand
Family2	*GhALDH2B1A*	Gh_A03G1229	A03	86766784–86770208	1179	392	42.64381	5.70	Chloroplast	minus
	*GhALDH2B2A*	Gh_A07G2011	A07	76376925–76380509	1596	531	57.63800	7.59	Mitochondrial	plus
	*GhALDH2B3A*	Gh_A12G1314	A12	69558172–69561824	1596	531	57.69817	6.81	Mitochondrial	plus
	*GhALDH2B4A*	Gh_A12G2471	A12	87183896–87187146	1623	540	58.81244	7.18	Mitochondrial	plus
	*GhALDH2B1D*	Gh_D02G1665	D02	57172439–57176663	1530	509	55.34062	6.80	Chloroplast	minus
	*GhALDH2B2D*	Gh_D07G2232	D07	53375582–53379207	1593	530	57.64695	8.03	Mitochondrial	plus
	*GhALDH2B3D*	Gh_D12G1438	D12	44221515–44225115	1596	531	57.74128	6.81	Mitochondrial	plus
	*GhALDH2B4D*	Gh_D12G2599	D12	58840207–58843432	1626	541	58.92050	7.18	Chloroplast	plus
	*GhALDH2C1A*	Gh_A05G0157	A05	1670874–1678965	1515	504	54.79314	8.33	Cytoplasmic	minus
	*GhALDH2C2A*	Gh_A06G0526	A06	10799343–10802205	1494	497	53.99529	7.11	Cytoplasmic	minus
	*GhALDH2C3A*	Gh_A07G0063	A07	761623–777944	2739	912	98.77655	5.97	Cytoplasmic	plus
	*GhALDH2C1D*	Gh_D05G0221	D05	2045263–2049124	1215	404	43.71334	7.05	Cytoplasmic	minus
	*GhALDH2C2D*	Gh_D06G0580	D06	9239274–9242178	1494	497	53.95119	7.11	Cytoplasmic	minus
	*GhALDH2C3D*	Gh_D07G0046	D07	498249–502320	933	310	33.44073	7.05	Cytoplasmic	minus
	*GhALDH2C4D*	Gh_D07G0047	D07	506235–511937	1548	515	55.99429	5.85	Cytoplasmic	minus
Family3	*GhALDH3F1A*	Gh_A02G1616	A02	82646608–82651284	1440	479	53.27214	8.62	PlasmaMembrane	minus
	*GhALDH3F2A*	Gh_A05G0568	A05	6073916–6077932	1470	489	54.74277	7.62	PlasmaMembrane	minus
	*GhALDH3F1D*	Gh_D03G0106	D03	792938–797752	1440	479	53.29011	8.66	PlasmaMembrane	plus
	*GhALDH3F2D*	Gh_D05G0697	D05	5668003–5672363	1473	490	54.81081	8.06	PlasmaMembrane	minus
	*GhALDH3H1A*	Gh_A02G0751	A02	14030633–14036873	1473	490	53.89183	8.27	Mitochondrial	minus
	*GhALDH3H2A*	Gh_A05G3716	scaffold1210_A05	27711–31024	1182	393	42.36826	8.81	PlasmaMembrane	minus
	*GhALDH3H3A*	Gh_A06G0384	A06	6429365–6435437	1473	490	53.62758	8.61	Mitochondrial	plus
	*GhALDH3H1D*	Gh_D02G0793	D02	12945983–12952298	1473	490	53.92185	8.27	Mitochondrial	minus
	*GhALDH3H2D*	Gh_D05G2245	D05	21555587–21560927	1482	493	54.20499	8.79	Chloroplast	plus
	*GhALDH3H3D*	Gh_D06G0414	D06	5912130–5918225	1473	490	53.69162	8.61	Mitochondrial	plus
	*GhALDH3I1A*	Gh_A05G3311	A05	86650719–86655533	1473	490	54.23894	6.15	Chloroplast	minus
	*GhALDH3I1D*	Gh_D04G0292	D04	4378806–4385236	1650	549	61.09512	8.65	Chloroplast	plus
Family5	*GhALDH5F1A*	Gh_A01G0799	A01	17510464–17544827	1530	509	54.67401	7.58	Chloroplast	minus
	*GhALDH5F1D*	Gh_D01G0827	D01	13131989–13153345	1653	550	59.69922	8.67	PlasmaMembrane	minus
Family 6	*GhALDH6B1A*	Gh_A03G1560	A03	96693079–96698709	1620	539	57.71440	8.23	Mitochondrial	minus
	*GhALDH6B2A*	Gh_A03G1561	A03	96700062–96712653	2478	825	90.58171	9.17	Mitochondrial	minus
	*GhALDH6B3A*	Gh_A10G0793	A10	16217188–16235086	3219	1072	117.91685	8.46	Nuclear	minus
	*GhALDH6B1D*	Gh_D02G2009	D02	64300173–64305816	1620	539	57.91762	8.61	Mitochondrial	minus
	*GhALDH6B2D*	Gh_D02G2010	D02	64307155–64315478	2118	705	77.40568	8.47	Cytoplasmic	minus
	*GhALDH6B3D*	Gh_D10G0970	D10	13151962–13169260	3405	1134	124.99664	8.94	Nuclear	plus
Family 7	*GhALDH7B1D*	Gh_D06G1578	D06	52817685–52822968	1524	507	54.33863	6.42	Chloroplast	minus
Family 10	*GhALDH10A1A*	Gh_A07G0563	A07	7809148–7814113	1512	503	54.69989	5.34	Chloroplast	minus
	*GhALDH10A2A*	Gh_A11G0380	A11	3483181–3488821	1509	502	54.75215	5.47	Cytoplasmic	minus
	*GhALDH10A1D*	Gh_D07G0629	D07	7306529–7311487	1512	503	54.72795	5.34	Chloroplast	minus
	*GhALDH10A2D*	Gh_D11G0441	D11	3702955–3708589	1509	502	54.71415	5.79	Cytoplasmic	minus
Family 11	*GhALDH11A1A*	Gh_A05G0415	A05	4681423–4683777	1491	496	53.16555	7.50	Cytoplasmic	plus
	*GhALDH11A2A*	Gh_A05G0479	A05	5198467–5201069	1494	497	53.28571	6.80	Cytoplasmic	plus
	*GhALDH11A3A*	Gh_A07G0209	A07	2502115–2504488	1500	499	53.48300	6.80	Cytoplasmic	plus
	*GhALDH11A1D*	Gh_D05G0533	D05	4326082–4328437	1491	496	53.17862	7.88	Cytoplasmic	plus
	*GhALDH11A2D*	Gh_D05G0594	D05	4784042–4786694	1494	497	53.17963	7.11	Cytoplasmic	plus
	*GhALDH11A3D*	Gh_D07G0263	D07	2751699–2754071	1500	499	53.44692	7.13	Cytoplasmic	plus
Family 12	*GhALDH12A1A*	Gh_A03G0575	A03	15033080–15037682	1665	554	61.51080	6.98	Mitochondrial	minus
	*GhALDH12A1D*	Gh_D03G0856	D03	29709339–29713965	1665	554	61.49283	7.27	Mitochondrial	minus
Family 18	*GhALDH18B1A*	Gh_A01G1899	A01	98932667–98937902	2196	731	79.25592	6.23	Mitochondrial	plus
	*GhALDH18B2A*	Gh_A04G1396	scaffold1000_A04	9715–18472	2193	730	79.16256	6.24	Mitochondrial	plus
	*GhALDH18B3A*	Gh_A10G2010	A10	97863671–97869528	2133	710	76.84524	6.40	Mitochondrial	minus
	*GhALDH18B4A*	Gh_A11G2925	A11	93106493–93111298	2211	736	78.73630	6.55	Mitochondrial	minus
	*GhALDH18B1D*	Gh_D01G2158	D01	60548378–60553524	2208	735	79.78740	6.53	Mitochondrial	plus
	*GhALDH18B2D*	Gh_D04G1204	D04	39407171–39416330	2193	730	79.17467	6.31	Mitochondrial	minus
	*GhALDH18B3D*	Gh_D10G2321	D10	61420676–61427664	2262	753	82.03947	6.61	Cytoplasmic	minus
	*GhALDH18B4D*	Gh_D11G3311	D11	65965823–65970213	2154	717	77.41345	6.09	Cytoplasmic	minus
Family 22	*GhALDH22A1A*	Gh_A02G0527	A02	7884791–7889318	1491	496	55.15361	6.58	PlasmaMembrane	plus
	*GhALDH22A1D*	Gh_D02G0592	D02	8046946–8052131	1788	595	65.95702	6.49	Cytoplasmic	plus

### Phylogenetic and structural analyses of upland cotton *ALDH* gene superfamily

**Fig 1:**
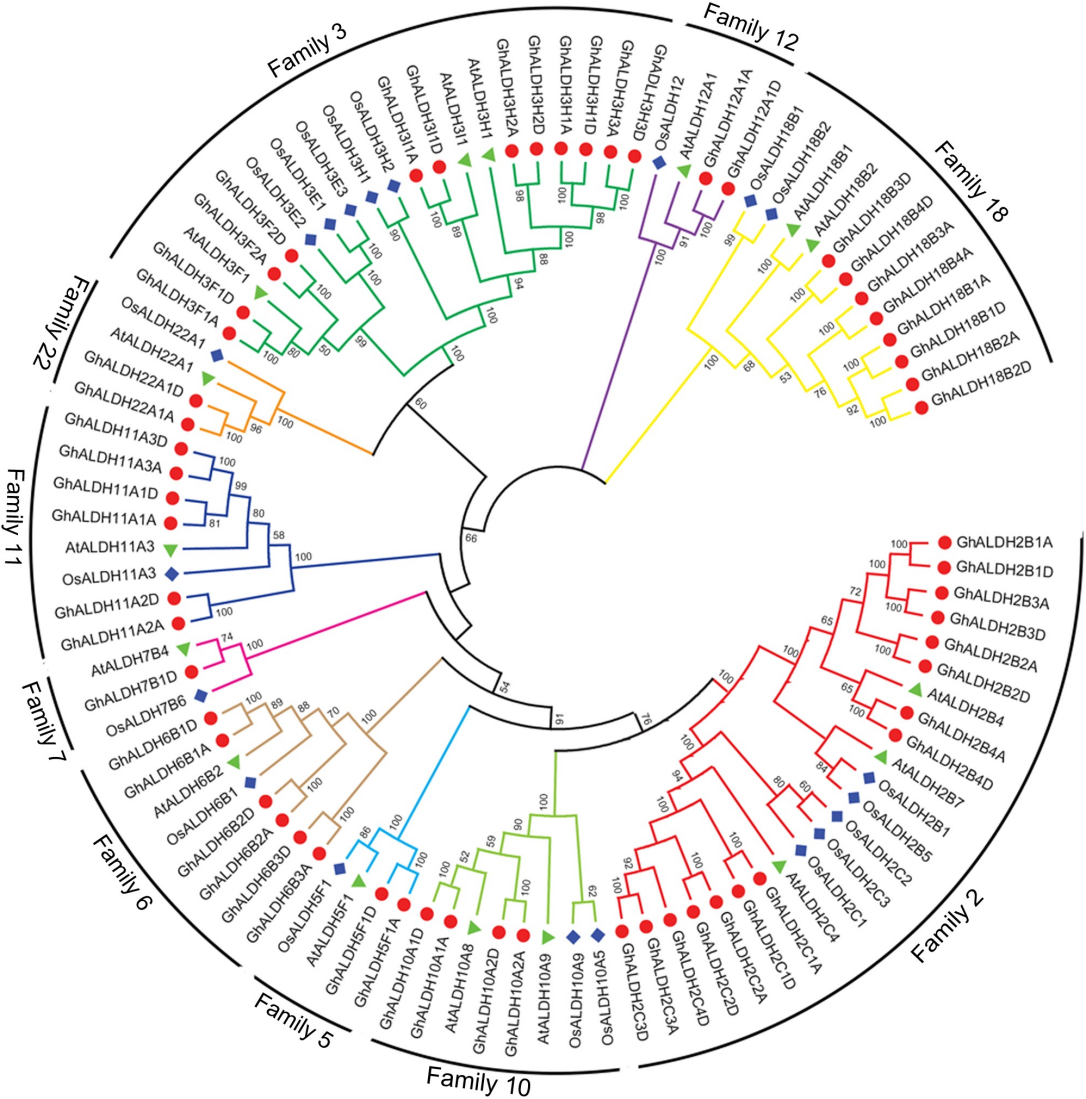
Phylogenetic analysis of the ALDH proteins from *G*. *hirsutum*, *Arabidopsis* and rice. The unrooted phylogentic tree was constructed using MEGA 5.2 by Neighbor-Joining method. Numbers on branches were bootstrap portions from 1000 replicates. Percentage bootstrap scores of <50% were hidden. The specific color indicated different families.

To assess the functional relevance of members of upland cotton *ALDH* gene superfamily, phylogenetic relationships among *G*. *hirsutum ALDHs* and other plant species was established. An unrooted phylogenetic tree derived from the ALDH amino acid sequences of *G*. *hirsutum*, *Arabidopsis* and rice was illustrated in Fig 1. The phylogenetic tree can be classified into 10 major groups which represented the 10 distinct ALDH protein families of *G*. *hirsutum*. In consistent with other previous studies, families 2, 5 and 10 grouped together, and families 3 and 22 were connected by a node, which belongs to the plant ALDH core families. Family 18 was the most phylogenetically distantly related ALDH family from the view of this topology. Meanwhile, an ML tree reconstructed with PhyML was almost consistent with the NJ tree except for minor differences at some branches (S1 Fig).

**Fig 2:**
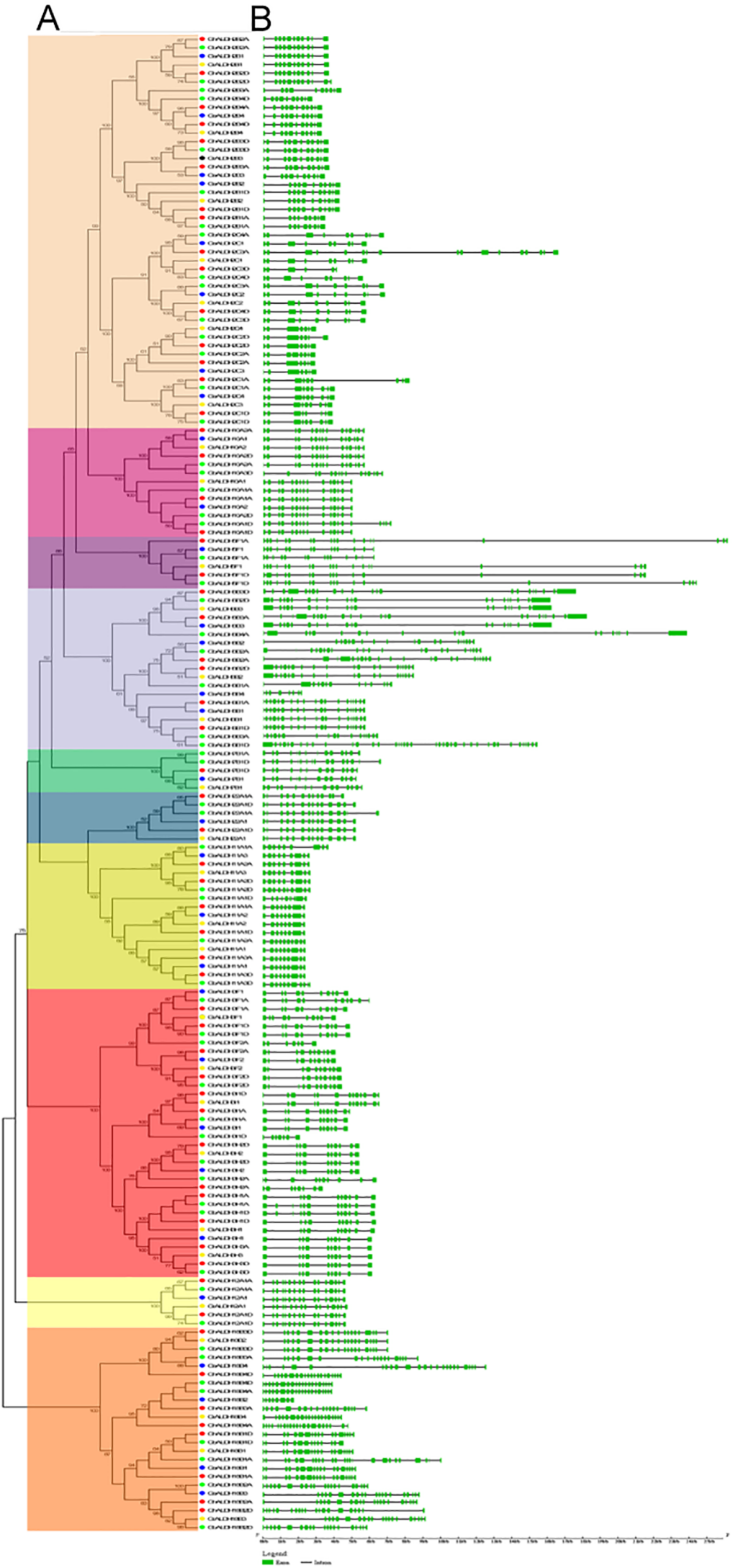
Phylogenetic relationships and gene structures of ALDHs from *G*. *arboreum*, *G*. *raimondii*, *G*. *hirsutum*, and *G*. *barbadense*. (A) The unrooted phylogenetic tree was constructed using MEGA 5.2 by Neighbor-Joining method and the bootstrap test was performed with 1,000 replicates. Percentage bootstrap scores of >50% were displayed. The colored shadow marks the different families of ALDH superfamily from the four surveyed cotton species. (B) Exon/intron structures of all the *ALDH* genes. The green boxes and gray lines respectively represented the exon and intron.

To obtain further insight into the evolutionary relationship among *G*. *hirsutum ALDH* gene superfamily and other three surveyed cotton species, all the putative ALDH proteins from *G*. *arboreum*, *G*. *raimondii*, *G*. *hirsutum* and *G*. *barbadense* were also aligned to construct an unrooted phylogenetic tree. As expected, the topology was similar to that generated with ALDH proteins from *G*. *hirsutum*, *Arabidopsis* and rice. As Fig 2A displayed, the core ALDH families 2, 5 and 10 clustered tightly, while family 18 was still the most phylogenetically distant. Form the view of evolution, one member of *ALDH* genes in diploid cottons would be correspondent with two homeologs from the A and D subgenomes of allotetraploid cotton species. In this study, most *ALDH* genes from *G*. *hirsutum* shown a one-to-one correspondence with those from its diploid progenitors, and the same phenomena was found in the other one cultivated allotetraploid cotton species *G*. *barbadense*. The inconsistencies including each a member loss of subfamilies 2B, 6B, and 11A in *G*. *barbadense*, and subfamilies 2C, 6B and 7B in *G*. *hirsutum*. Furthermore, one more putative *ALDH* gene was present in the ALDH10A subfamily of *G*. *barbadense* and ALDH3H subfamily of *G*. *hirsutum* respectively. In particular, the homologous genes were almost in the terminal branches of the phylogenetic tree with high bootstrap values. And those genes within the same subfamilies from the same subgenome of allotetraploids tended to cluster together, suggesting close relationship between them. Surprisingly, *ALDH* genes from the A subgenome and D subgenome of *G*. *hirsutum* shown a bias to those from *G*. *arboreum* and *G*. *rainondii*. Meanwhile, the phylogenetic tree reconstructed with ML method was almost consistent with the one of NJ method except for minor differences at some branches (S2 Fig), which validated the reliability of our results.

The genomic structures was vital to reveal the evolutionary history within *ALDH* gene families. We compared the *ALDH* gene structures and found that genes from the same subfamily usually possessed a highly conserved exon-intron organization within and across the four surveyed cotton species (Fig 2B). In *G*. *hirsutum*, the numbers of exons of *ALDH* genes varied from six to 22. Some *ALDH* genes even shared identical number and length of exon such as genes from subfamilies 2B, 2C, 10A, 11A, and 12A. Such conserved gene structures within each subfamily indicated that cotton *ALDH* genes have underwent duplication events during evolution. Compared with the *ALDH* genes of intra-species, the *ALDH* genes from the A and D subgenomes of the two allotetraploid cottons were more similar to those from their ancestor species respectively. Even so, exon gains or losses still occurred during evolution with subfamily 22A as an example. *GaALDH22A1*, *GrALDH22A1*, *GhALDH22D1*, and *GbALDH22D1* each contained 14 exons, while *GhALDH22A1A* possessed 13 exons and *GbALDH22A1A* had 15 exons.

### Chromosomal distribution and expansion patterns of upland cotton *ALDH* gene superfamily

**Fig 3:**
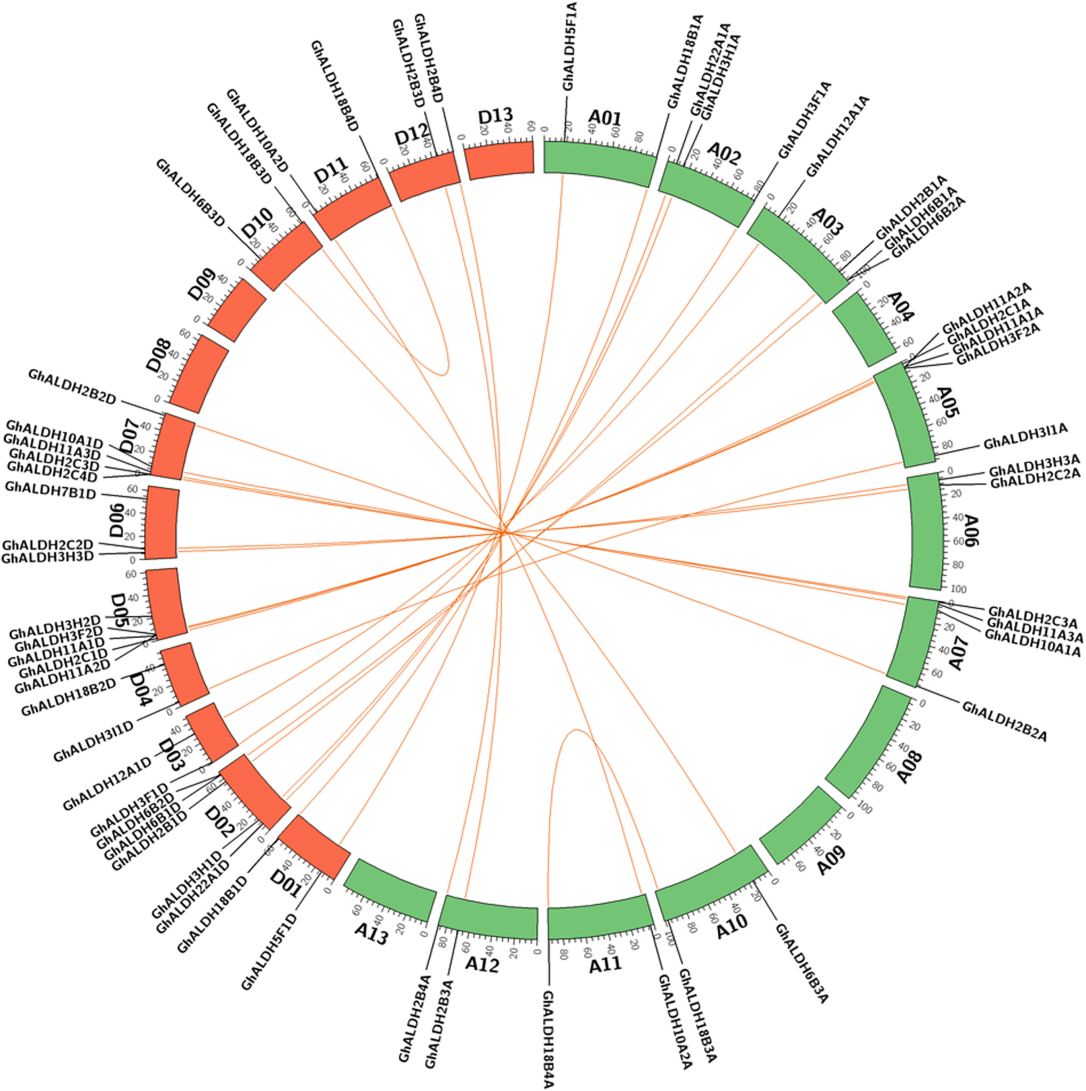
Chromosome distribution and gene duplication of *GhALDH* gene superfamily. The picture was generated by Circos software. The chromosomes of A-subgenome and D-subgenome from *G*. *hirsutum* were shown with different colors and labeled as A or D followed by corresponding numbers, respectively. The duplicated gene pairs were connected with orange lines.

The mapping of the gene loci shown that *ALDH* genes were distributed unevenly on 19 of 26 *G*. *hirsutum* chromosomes. As illustrated in Fig 3, Chr A05, D02, D05 and D07 contained five *ALDH* genes each, followed by Chr A03 and A07 on which four *ALDH* genes were located. Additionally, *GhALDH3H2A* and *GhALDH18B2A* were distributed on the scaffolds related to Chr A05 and A04, respectively. The remaining genes were dispersed on other chromosomes.

To examine the driving force for gene evolution, the nonsynonymous and synonymous substitution (dN and dS) of duplicated genes were calculated using the full-length sequences. A dN/dS ratio of 1 was set as a cut-off value for identify genes under negative selection. As demonstrated in Table 3, almost all the duplicated gene pairs were likely under purifying selection pressure with the dN/dS ratio < 1, except for *GhALDH3F2A*/*GhALDH3F2D*, suggesting that the two genes had experienced positive selection.

**Table 3:** dN/dS analysis for the duplicated *ALDH* gene pairs in *G*. *hirsutum*.

Duplicated gene 1	Duplicated gene 2	dN	dS	dN/dS
*GhALDH2B1A*	*GhALDH2B1D*	0.0298	0.0551	0.5407
*GhALDH2B2A*	*GhALDH2B2D*	0.0063	0.0404	0.1554
*GhALDH2B3A*	*GhALDH2B3D*	0.0088	0.0319	0.2749
*GhALDH2B4A*	*GhALDH2B4D*	0.0051	0.0465	0.1107
*GhALDH2C1A*	*GhALDH2C1D*	0.0137	0.0673	0.2040
*GhALDH2C2A*	*GhALDH2C2D*	0.0056	0.0153	0.3692
*GhALDH2C3A*	*GhALDH2C3D*	0.0181	0.0508	0.3560
*GhALDH2C3D*	*GhALDH2C4D*	0.0564	0.226	0.2494
*GhALDH3F1A*	*GhALDH3F1D*	0.0139	0.0496	0.2803
*GhALDH3F2A*	*GhALDH3F2D*	0.0145	0.0061	2.3895
*GhALDH3H1A*	*GhALDH3H1D*	0.0010	0.0431	0.0224
*GhALDH3H2A*	*GhALDH3H2D*	0.0091	0.0182	0.5000
*GhALDH3H3A*	*GhALDH3H3D*	0.0056	0.0393	0.1416
*GhALDH3I1A*	*GhALDH3I1D*	0.0106	0.0263	0.4025
*GhALDH5F1A*	*GhALDH5F1D*	0.0341	0.0649	0.5254
*GhALDH6B1A*	*GhALDH6B1D*	0.0085	0.0369	0.2314
*GhALDH6B2A*	*GhALDH6B2D*	0.0100	0.0388	0.258
*GhALDH6B3A*	*GhALDH6B3D*	0.0196	0.0443	0.4422
*GhALDH10A1A*	*GhALDH10A1D*	0.0039	0.0187	0.2062
*GhALDH10A2A*	*GhALDH10A2D*	0.0068	0.0209	0.3258
*GhALDH11A1A*	*GhALDH11A1D*	0.0047	0.0155	0.3015
*GhALDH11A3A*	*GhALDH11A3D*	0.0055	0.0357	0.1526
*GhALDH12A1A*	*GhALDH12A1D*	0.0049	0.0403	0.1222
*GhALDH18B1A*	*GhALDH18B1D*	0.0195	0.0621	0.3149
*GhALDH18B2A*	*GhALDH18B2D*	0.0036	0.0406	0.0883
*GhALDH18B3A*	*GhALDH18B4A*	0.1149	1.8984	0.0605
*GhALDH18B3D*	*GhALDH18B4D*	0.1159	2.0883	0.0555
*GhALDH22A1A*	*GhALDH22A1D*	0.0054	0.0407	0.1333

### Expression profiles of upland cotton *ALDH* gene superfamily under salt stress

**Fig 4:**
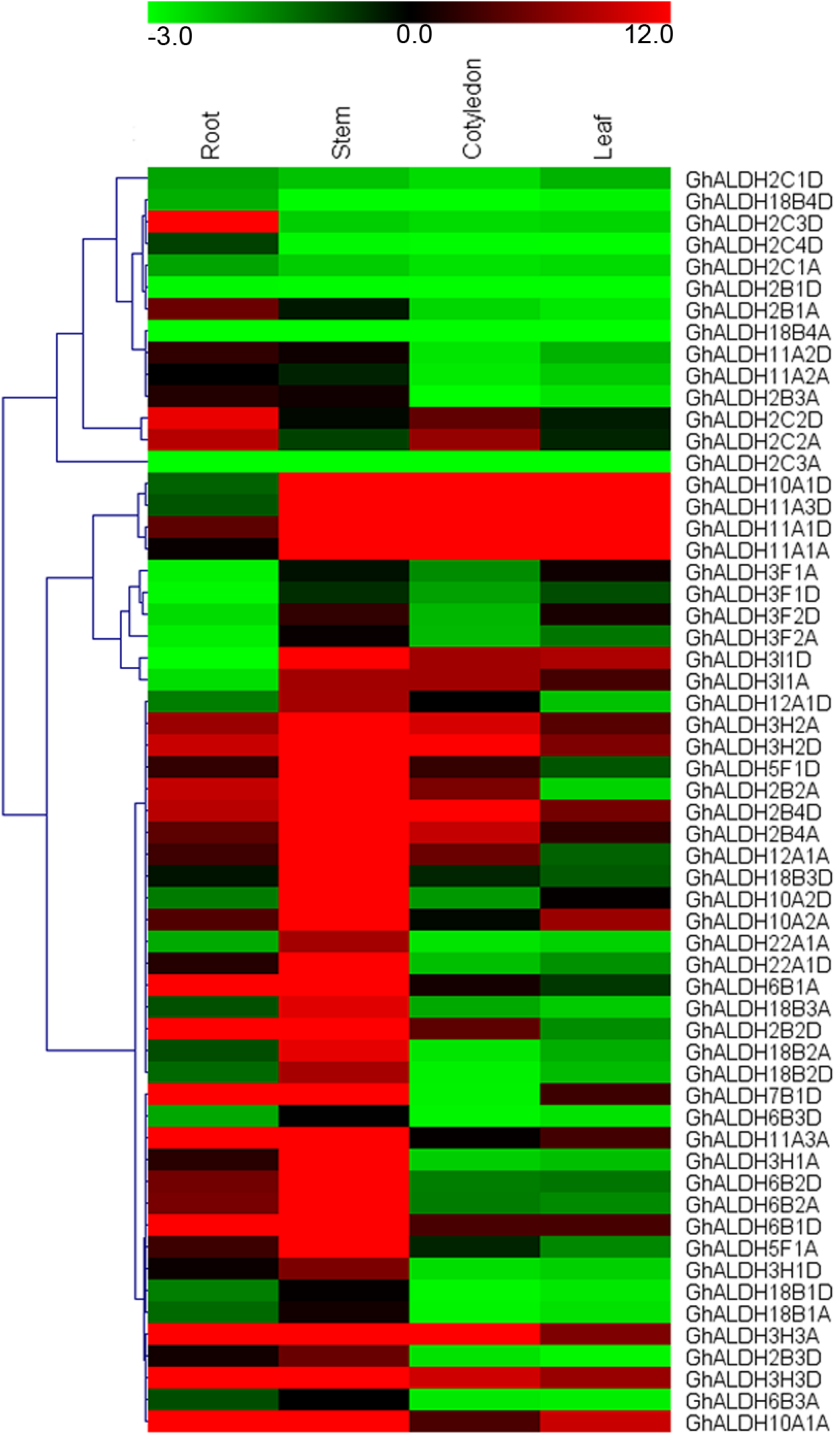
Expression profiles of *GhALDH* gene superfamily in four representative tissues of *G*. *hirsutum*. The heat map shows the real-time quantitative RT-PCR (q-RT-PCR) analysis results of *GhALDH* genes in Upland cotton TM-1. The colour bar represents the relative signal intensity values.

A comprehensive qRT-PCR analysis was performed to obtain the expression patterns of *ALDH* gene superfamily in *G*. *hirsutum*. As displayed in Fig 4, most ALDH-encoding genes showed predominant expression in roots and stems compared with cotyledons and leaves. In most cases, the genes from the same family with conserved structure didn’t cluster together, suggesting a function divergence during evolution. Most *GhALDH* genes shown a tissue-specific expression pattern with the exception of *GhALDH3H2A*/*GhALDH3H2D*, *GhALDH3H3A*/*GhALDH3H3D*, and *GhALDH10A1A* which exhibited abundant in all the tissues detected. Notably, for *GhALDH10A1D*, *GhALDH11A1A*/*GhALDH11A1D*, and *GhALDH11A3D* genes, high level accumulation existed in stem, cotyledon, and leaf, but not in root. On the contrary, the expression level of *GhALDH2C1A*/*GhALDH2C1D*, *GhALDH18B4A*/*GhALDH18B4D*, and *GhALDH2C3A* couldn’t be detected almost in all the four tissues surveyed.

**Fig 5:**
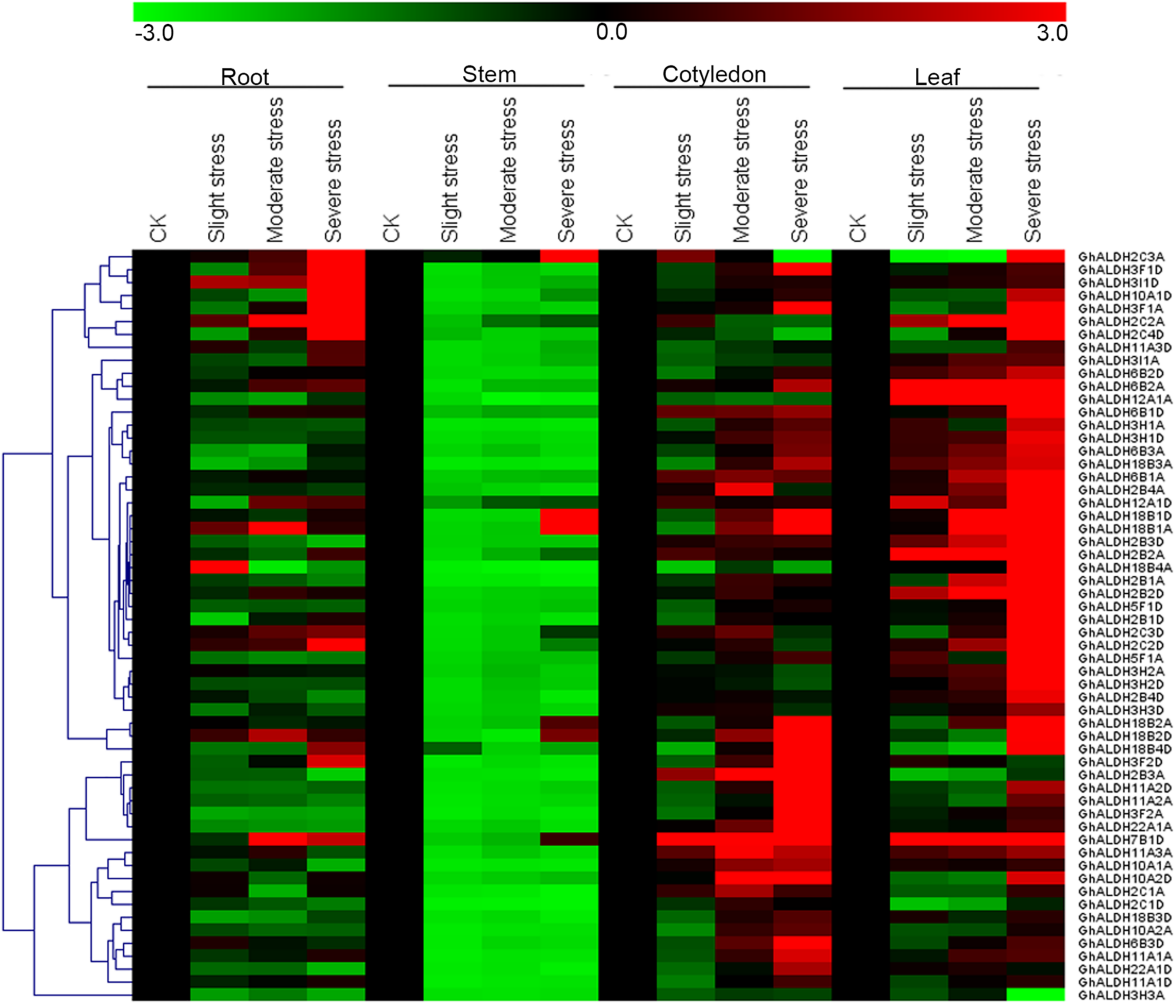
Expression profiles of *GhALDH* gene superfamily in four representative tissues of *G*. *hirsutum* under salt stress. The heat map shows the real-time quantitative RT-PCR (q-RT-PCR) analysis results of *GhALDH* genes in Upland cotton TM-1 with salt treatments. The slight stress, moderate stress, and severe stress represents 100 mM, 150 mM and 200 mM NaCl, respectively. The colour bar represents the relative signal intensity values.

Researches have shown that the plant *ALDH* genes were involved in a wide range of stress response pathways. Therefore, we particularly aim at the expression pattern changes of *ALDH* gene superfamily under salt stress in upland cotton. The heatmap of *G*. *hirsutum ALDH* gene superfamily expression profile under salt stress was presented in Fig 5. The relative expression levels of *GhALDHs* under salt treatment differed among each subfamilies. A majority of *ALDH* genes shown altered expression patterns of either induction or suppression associated with at least one salt treatment. In roots and stems, nearly no *ALDH* genes were induced under salt stress except for *GhALDH2C3A*/*GhALDH2C3D* and *GhALDH18B1A*/*GhALDH18B1D*. Transcripts of *GhALDH18B4A* was initially increased under a slight salinity conditions and then dropped under severe salinity conditions in roots. Fifty-four *GhALDH* genes shown an up-regulated expression trend in leaves in response to seriously salt treatments. By contrast, the number of up-regulated *ALDH* genes in cotyledons were less. In leaves, *GhALDH6B2A*, *GhaLDH12A1A*, *GhALDH2B2A*, and *GhALDH7B1D* presented a continuous increase of transcript accumulation under the salt treatments. And *GhALDH2B3A*, *GhALDH2C1D*, and *GhALDH3H3A* were down-regulated under stress condition.

## Discussion

### The phylogenetic relationship of *ALDH* gene superfamily were highly related among two allotetrapoliod cotton sepcies and their diploid progenitors

The releases of genome assembly for four *Gossypium* species makes it easy to analyze the stress response related gene families through comparative genomics method [17–22]. In this study, putative ALDH sequences belonging to 10 ALDH families were individually identified in the genomes of *G*. *arboreum*, *G*. *raimondii*, *G*. *hirsutum*, and *G*. *barbadense*. Compared with other known plant ALDH superfamilies such as *Arabidopsis* [5], rice [6], and *populus* [8], *Gossypium* possessed the most expanded ones, with 30 members in both diploid species and 58 in both allotetraploid species, respectively. A previous studies identified 30 *G*. *raimondii ALDH* genes based on the same genome data we used. We checked the result by BLASTP and tBlastN and found to be consistent. From the theory of evolution, one single *ALDH* gene in diploid cottons should be corresponding to two homeologs in their allotetraploid derivatives. However, the numbers of *ALDH* genes in *G*. *hirsutum* and *G*. *barbadense* were less than the total sum of those from *G*. *arboreum* and *G*. *raimondii*, not twofold theoretically. This could be explained by that gene loss during the evolution of allotetraploid cottons after speciation. In addition, the size of *ALDH* gene superfamily is the same in two diploid cottons, albeit the genome of *G*. *arboreum* is approximately twice larger than that of *G*. *raimondii*. This may be associated with the long terminal repeat (LTR) retrotransposons insertion along each chromosome in *G*. *arboreum* [17–19]. However, the relatively same size of *ALDH* gene superfamily among the four surveyed cotton species reflects the high conservation of *ALDH* genes during evolution.

In order to reveal the homologous relationships of *ALDH* gene superfamily among different taxa, a phylogenetic tree was generated with full-length ALDH proteins from *G*. *hirsutum*, *Arabidopsis*, and rice. As Fig 1 illustrated, GhALDHs were more closely related with AtALDHs than OsALDHs, which was consistent with the evolutionary relationships among the three species. The topology of the other phylogenetic tree constructed with full-length ALDH proteins from the four surveyed cottons was similar to that mentioned above. The two phylogenetic trees indicated that the plant core ALDH families 2, 5, and 10 were grouped together. And family 18 was the most distantly related one, which was similar to that from other plant species such as *populus* [8], grape [42], and *P*. *trichocarpa* [43]. Meanwhile, our results have complemented the earlier study of ALDH superfamily in *G*. *raimondii* [15] by the comparative genomics approach. Furthermore, it’s worth noting that families 5, 7, 12, 22 were represented by only one gene number in all the surveyed diploid species, and one or two counterparts in allotetraploids cottons. It is speculated that these families may act as ‘house-keeping genes’ to participate in the fundamental metabolism and physiological pathways of plants to keep balance of aldehyde concentration. In contrast, family 2 and family 3 are the two most expanded groups in the six plant species we investigated. Studies shown that the *ALDH2* gene family can degrade the acetaldehyde generated through ethanolic fermentation [44,45]. In *Arabidopsis*, *ALDH3I1* expression only can be detected in leaves and induced by stress treatments such as ABA exposure, salinity, dehydration, heavy metals, oxidants and pesticides [46,47]. The expansion of *ALDH2* and *ALDH3* gene families compared with other families suggested that these *ALDH* genes may be essential for plants to cope with environmental stresses. Additionally, the *ALDH* gene members from the subgenomes of two allotetraploid cottons were more phylogenetic closely to their diploid genome ancestors. It reflected that ALDH superfamily evolved before the formation of allotetraploid cotton species by a polyploidization event.

### The *ALDH* gene superfamily were greatly conserved in four *Gossypium* species

To explore evolutionary characters of *ALDH* genes among diploids and allotetraploids, we directly compared the gene structures of different species. A high level of structural identity was observed among the *ALDH* genes from the same subfamily. The conservation of gene structures correlated well with the phylogetic clades. Such phenomena indicated that cotton *ALDH* gene superfamily have underwent duplication events during evolution. Also, the *ALDH* gene members from the A-subgenome and D-subgenome of allotetraploid cottons were structurally more similar to those of its A- and D-genome progenitor, separately. It further supported the topology of our phylogenetic tree. Meanwhile, this might be a result from genome duplication occurrence earlier than segmental duplication. Gene duplication events, including tandem duplication, segmental duplication, transposition events, and whole-genome duplication, are the major reason for the amplification of gene family [48,49]. In *G*. *hirsutum*, whole-genome duplicaton mainly contributed to the expansion of *ALDH* gene superfamily. And an intriguing finding was that purifying selection predominated across the duplicated genes. A likely reason for this observation is that, for a new duplicate gene, deleterious loss-of-function mutations were tended to happen. However, purifying selection could eliminate it, thus fixed the retention in a genome and function of both duplicate loci [50–52].

### *ALDH* gene superfamily shown a functional diversity in response to salt stress in *G*. *hirsutum*

The gene expression patterns can provide important clues for gene function. Our qRT-PCR results demonstrated the different expression patterns of *ALDH* gene superfamily across tissues of upland cotton. The conservation of *ALDH* gene superfamily in plants implied their significance in fundamental processes. There must exist strong selective pressure to maintain the gene function. Functional analyses of most *ALDH* genes shown that they shared the same stress response pattern among various plants [5]. In the study, a majority of *GhALDH* genes were up-regulated in leaves under severe salt stress, although roots are the tissues directly exposing to environmental stresses. This may be associated with the facts that these two tissues by themselves were distinct in structure and functions [53].

*Arabidopsis ALDH* gene *AtALDH10A9* was reported to be weakly induced by different abiotic stressors [54]. Transgenic *Arabidopsis* plants overexpressing *AtALDH7B4* were more tolerant to salt stress and show reduced accumulation of malondialdehyde (MDA) in comparison to the wild-type ones [55]. Analogously, the expression of orthologous gene *GhALDH10A2D* and *GhALDH7B1D* were induced significantly in leaves under salt stress in our study. In addition, most of the duplicated gene pairs demonstrated a high degree of functional divergence in response to salt treatment. In leaves, *GhALDH2B3A* shown a high level of accumulation in response to salt stress, while the closely-related gene *GhALDH2B3D* shown down-regulated. It could be explained by the assertion that the duplicated genes always underwent massive silencing and elimination after whole-genome duplication. This has long been recognized as a pervasive force in plant evolution [56]. Nonfunctionalization, subfunctionalization, and neofunctionalization are the three evolutionary fates of duplicate genes. And the functional divergence among duplicate genes can increase their chance to be retained in a genome [57]. What’s more, the expression level dominance exhibited by *G*. *hirsutum* under salt stress was unbalanced toward the D-subgenome, more *ALDH* genes (27 of 30 *ALDH* genes) were induced than that of A-subgenome (23 of 28 *ALDH* genes) in leaves. This is consistent with the nature of its diploid ancestors, i.e., in the genomic group of A-genome progenitor, long fiber first involved; while in the D-genome parent, the feature of adaptation to adverse environmental stresses was dominant.

## Conclusions

A comparative genomics approach was carried out to investigate ALDH superfamily in upland cotton. The phylogenetic relationships and gene structure were evaluated in the four cotton species, *G*. *arboreum*, *G*. *raimondii*, *G*. *hirsutum*, and *G*. *barbadense*. The tissue-specific expression profiles of *GhALDH* gene superfamily were detected. Future work will reveal the physiological role of different *ALDH* genes in dealing with abiotic stress in *Gossypium* species. Our findings may provide a framework to understand the evolution of *ALDH* gene superfamily in plants and help in the identification of key genes which can be used in the improvement of salt tolerance for cotton.

## Supporting information

S1 Fig Phylogenetic relationships of ALDH proteins from *G*. *hirsutum*, *Arabidopsis* and rice. The unrooted phylogentic tree was constructed using PhyML software by Maximum Likelihood method with LG model. The bootstrap test was performed with 1,000 replicates. Different ALDH families were represented by specific colors.
(TIF)

S2 Fig Phylogenetic relationships of ALDH proteins from *G*. *arboreum*, *G*. *raimondii*, *G*. *hirsutum*, and *G*. *barbadense*. The unrooted phylogentic tree was constructed using PhyML software by Maximum Likelihood method with LG model. The bootstrap test was performed with 1,000 replicates. Different ALDH families were represented by specific colors.
(TIF)

S1 Table Gene-specific primers for q-RT-PCR used in this study. (XLSX)

S2 Table The information of *ALDH* genes in *Gossypium*. (XLSX)
