# Supplementary figures and images for: Comparative genomic study of ALDH gene superfamily in Gossypium: A focus on Gossypium hirsutum under salt stress
Source: PLoS One. 2017 May 10;12(5):e0176733. doi: 10.1371/journal.pone.0176733 (PMC5425181; doi:10.1371/journal.pone.0176733)

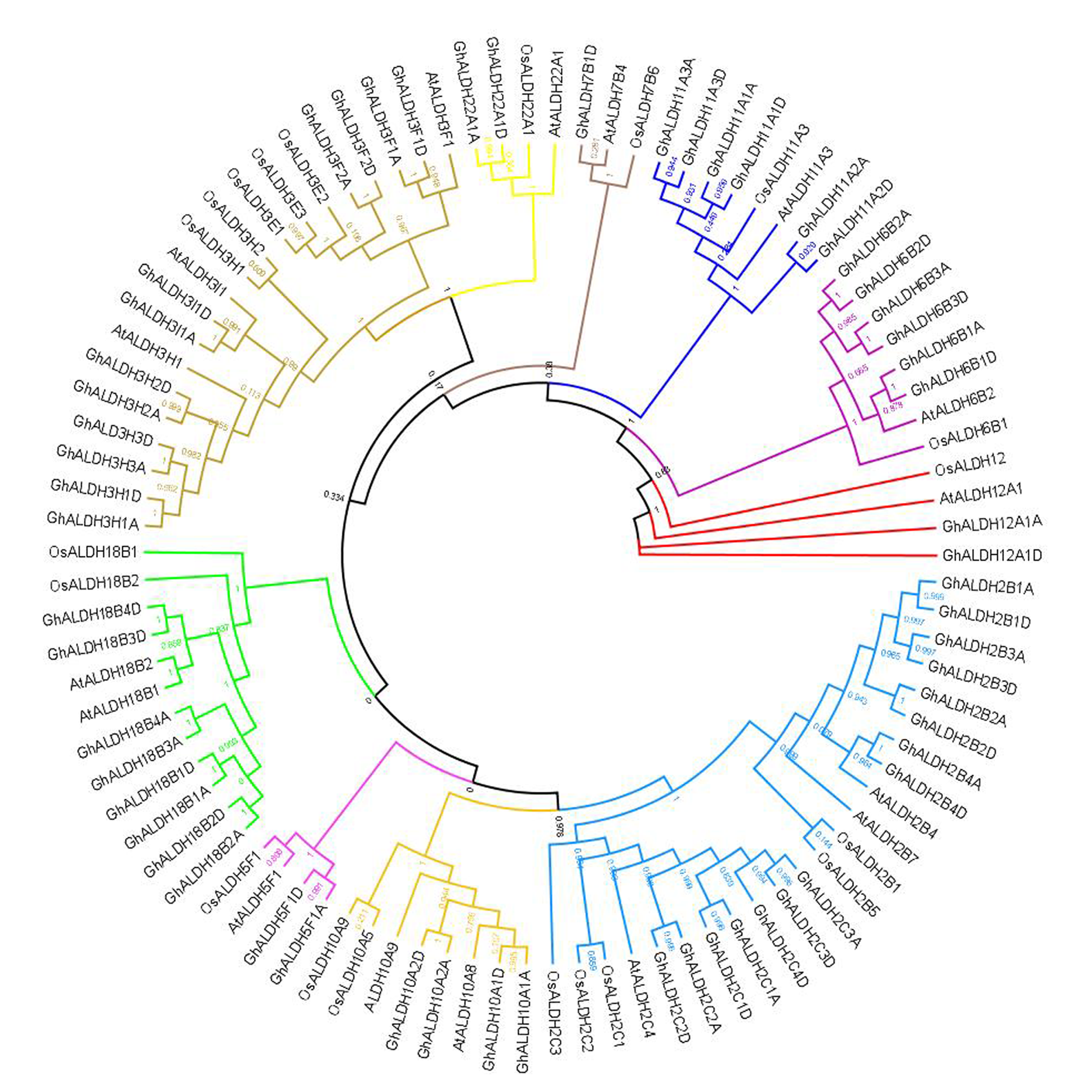

Supplement: S1 Fig — The unrooted phylogentic tree was constructed using PhyML software by Maximum Likelihood method with LG model. The bootstrap test was performed with 1,000 replicates. Different ALDH families were represented by specific colors. (TIF) [file pone.0176733.s001.tif]

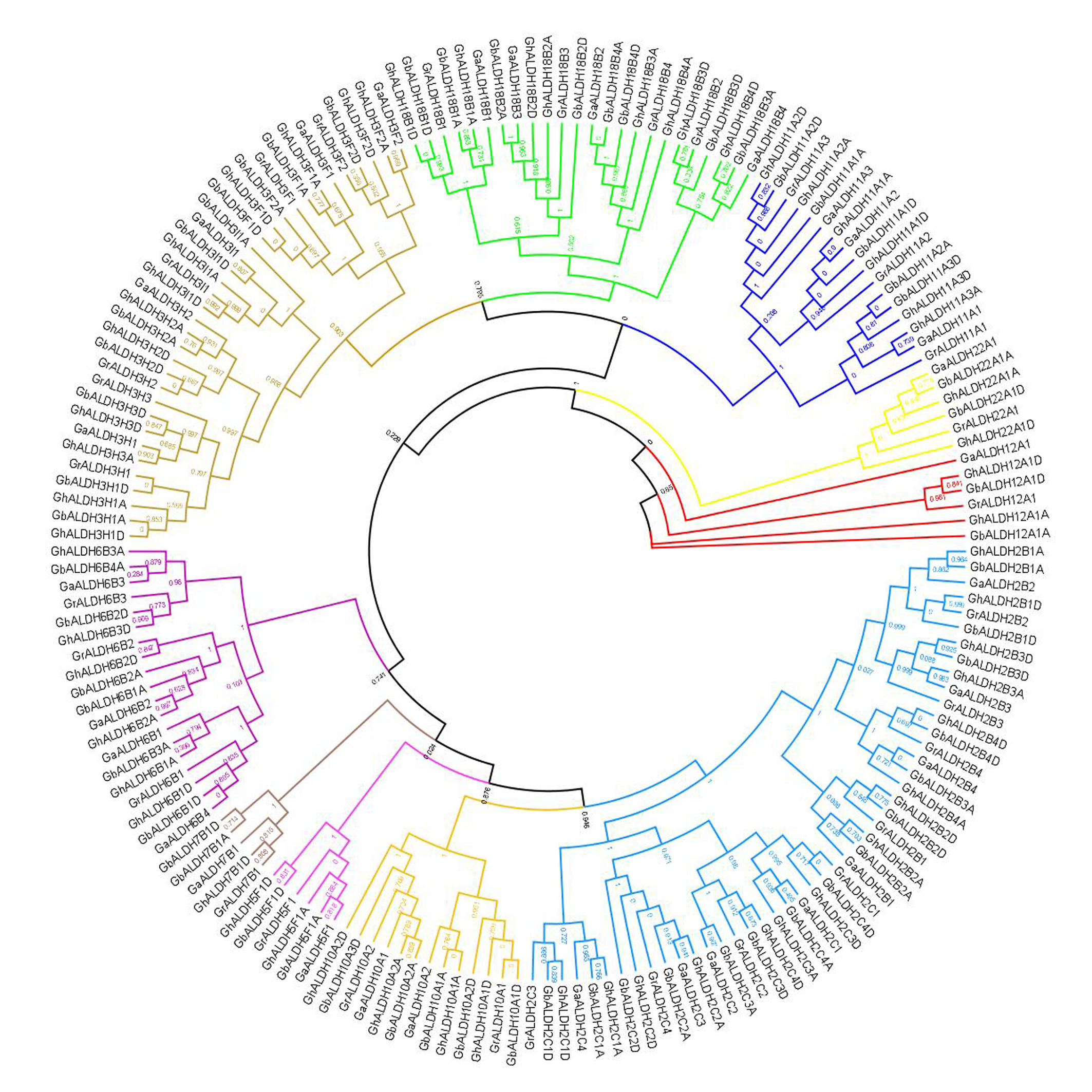

Supplement: S2 Fig — The unrooted phylogentic tree was constructed using PhyML software by Maximum Likelihood method with LG model. The bootstrap test was performed with 1,000 replicates. Different ALDH families were represented by specific colors. (TIF) [file pone.0176733.s002.tif]
